# Long-term interleukin-4 release from 3D printable affinity hydrogels promotes M2-like macrophage polarisation *in vitro*†

**DOI:** 10.1039/d4bm01623h

**Published:** 2025-03-28

**Authors:** George A. Loxley, Consuelo Coser, Amir M. Ghaemmaghami, Jing Yang

**Affiliations:** a Division of Regenerative Medicine and Cellular Therapies, Biodiscovery Institute, School of Pharmacy, University of Nottingham Nottingham NG7 2RD UK jing.yang@nottingham.ac.uk; b Immunology & Immuno-bioengineering Group, School of Life Sciences, Faculty of Medicine and Health Sciences, University of Nottingham Nottingham NG7 2RD UK amir.ghaemmaghami@nottingham.ac.uk; c Biodiscovery Institute, School of Pharmacy, University of Nottingham Nottingham NG7 2RD UK; d Section of Immunology, School of Biosciences, University of Surrey Guildford Surrey GU2 7XH UK

## Abstract

The biopharmaceutical industry for engineered protein drugs is rapidly increasing in size but there is a lack of controlled release vehicles to enable targeted delivery for regenerative medicine applications. In this study, we used photocrosslinkable 3-sulfopropyl acrylate potassium salt (SPAK)–poly(ethylene glycol) diacrylate (PEGDA) hydrogels to achieve controlled release of lysozyme for 70 days with zero-order release and tuneable release rate. Scaling down hydrogel volume and protein loading concentration to release Transforming growth factor beta-1 (TGF-β1) and Interleukin-4 (IL-4) resulted in low cumulative release, even without SPAK. Increasing PEGDA molecular weight from 4 kDa to 20 kDa improved TGF-β1 release but it still remained below 10% after 10 days. We observed sustained IL-4 release in the therapeutic ng mL^−1^ range for 73 days when loading IL-4 to 5% SPAK–10% PEGDA post photocrosslinking. Released IL-4 maintained bioactivity, promoting M2-like polarisation of THP-1 macrophages with day 53 supernatant, modelling long-term immunomodulation *in vitro*. We manufactured SPAK–PEGDA hydrogels by projection micro stereolithography, in which 3D printed 5% SPAK–10% PEGDA had an increased lysozyme release rate compared to its cast counterpart. 3D printed 5% SPAK–10% PEGDA with porous 3D design had an increased lysozyme release rate compared to a volume matched non-porous design. These findings highlight the potential of SPAK–PEGDA hydrogels for long-term cytokine delivery and show proof-of-concept for manipulating protein release kinetics with 3D printed hydrogel design.

## Abstract 2

Localised cytokine delivery is a promising strategy for modulating immune response and enhancing regenerative medicine therapies. SPAK-PEGDA hydrogels enable the long-term controlled release of lysozyme and the sustained release of Interleukin-4.

## Graphical abstract

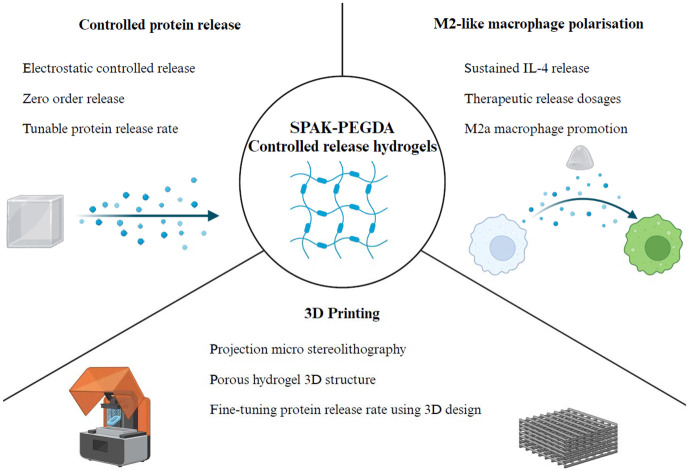

## Introduction

The biopharmaceutical market for engineered protein drugs was worth approximately $400 billion in 2022 and is predicted to rapidly increase to over $650 billion by 2030.^1,2^ Among these proteins, cytokines are of particular interest because of their high potency, specificity, and natural role in biological signalling.^3^ Despite years of research and development, the only recombinant cytokine treatments with FDA approval in the regenerative medicine field are bone morphogenic protein 2 (BMP-2) and platelet derived growth factor (pdGF).^4^

Difficulties in product generation arise due to the short half-life of cytokines *in vivo*, their susceptibility to denaturation, and the lack of targeted delivery methods.^5,6^ The controlled release of cytokines from implanted medical devices is the ideal delivery method because it avoids the off target side effects observed in systemic administration and prevents the need for repeat injections at the target site. Infuse™, a BMP-2 release product for spinal regeneration, had severe adverse effects at clinical trials in which ectopic bone growth, inflammation, and nerve damage occurred due to the release of supraphysiological doses of BMP-2.^7,8^ Such negative outcomes highlight the requirement for control over cytokine release rate and concentration so that target cells are exposed to physiologically relevant cytokine doses for clinically relevant time periods.

Hydrogels are promising vehicles for protein delivery due to their ability to absorb water and generate a swollen network promoting protein stability.^9^ Few experimental reports have shown *in vitro* protein release from hydrogels for multiple weeks or months which is the time frame for regenerative therapies.^10,11^ Incorporation of hydrolysable mercaptoacetic ester linkages enabled the release of bovine serum albumin (BSA) from poly(ethylene glycol) diacrylate (PEGDA) hydrogels for 60 days and basic-fibroblast growth factor for 35 days.^12^ Similarly, addition of thiol residues into photocrosslinkable dextran hydrogels resulted in delayed release of IgG after 8 months.^13^ The main limitations of these approaches are that material hydrolysis is modelled in simplified *in vitro* conditions that may not represent degradation *in vivo* and that protein bioactivity is often not assessed after extended periods of release.^13–17^

Electrostatic release methods have traditionally utilised binding affinity between glycosaminoglycans (GAGs) and proteins to control protein release rate. Heparin has been most commonly used due to its ability to sequester heparin binding proteins to prolong their half-life *in vivo*.^18^ Alginate methacrylate hydrogels containing methacrylated heparin controlled the release rate of BMP-2, Transforming growth factor beta-1 (TGF-β1), Fibroblast-like growth factor 2 (FGF-2), and Vascular endothelial growth factor (VEGF) for 3 weeks in comparison to heparin-free alginate hydrogels.^19^ Sulfated alginate was produced as an animal product free alternative to heparin and originally controlled the release of basic FGF for a 5-day period.^20^ More recently, sulfated alginate has been used to control the release of BMP-2 for 10 days from bioprinted bone tissue engineering scaffolds.^21^ Affinity release methods have generally shown short-term release data compared to controlled degradation, and long-term protein release studies have often not reported if the released concentration is within therapeutic ranges.^13–15,19,21^

Macrophages can orchestrate immune signalling in pro-inflammatory or anti-inflammatory directions based on their polarisation state and are a therapeutic target in multiple regenerative medicine applications. Polarisation exists on a spectrum with the historic poles of M1 and M2 respectively.^22,23^ Anti-inflammatory M2-like polarisation is promoted by Interleukin-4 (IL-4), Interleukin-13 (IL-13), Interleukin-10 (IL-10), and transforming growth factor beta 1 (TGF-β1) which makes it an ideal target for controlled cytokine delivery.^24–27^ Promoting M2-like macrophages has been a strategy to prevent foreign body response to tissue engineering scaffolds,^28^ enhance osteogenesis in bone regeneration,^29^ and to reduce pro-inflammatory signalling in osteoarthritic joints.^30^

3D printing has gained much attention in the regenerative medicine field for the production of materials with application-specific geometries and fine control over architecture. In controlled release, paracetamol tablets have been printed by stereolithography with varying surface area to volume ratios to influence drug elution rate.^31^ Whether 3D printed hydrogel design may be used to optimise protein release remains unexplored.

In the present study we used photocrosslinkable 3-sulfopropyl acrylate (SPAK)-PEGDA hydrogels to control the release of lysozyme for 70 days. We hypothesized that hydrogel volume and loading concentration could be scaled down to achieve similar long-term controlled release of M2 promoting cytokines in therapeutic dosages. A series of optimisation experiments were performed to improve the low cumulative release initially seen in TGF-β1 and IL-4 from PEGDA. IL-4 release was sustained in the ng mL^−1^ concentration range for 73 days when loaded in SPAK–PEGDA hydrogels post photocrosslinking. IL-4 bioactivity was assessed by promoting M2-like polarisation of THP-1 macrophages. SPAK–PEGDA hydrogels were 3D printed by projection micro stereolithography and the release of lysozyme was compared between a porous and non-porous 3D design over a 15-day period.

## Materials and methods

### Materials

The following materials were purchased from Sigma Aldrich: Dulbecco's Phosphate Buffered Saline (PBS), 2-Hydroxy-4′-(2-hydroxyethoxy)-2-methylpropiophenone (Irgacure-2959), Lithium phenyl-2,4,6-trimethylbenzoylphosphinate (LAP), PEGDA (Mn 575 Da, 4 kDa, 6 kDa, and 20 kDa), SPAK, Lysozyme from chicken egg whites, Micrococcus lysodeikticus, Bovine serum albumin (BSA), Human recombinant IL-4, Foetal bovine serum (FBS), Penicillin–streptomycin, Phorbol 12-myristate 13-acetate (PMA), Macrophage Colony-Stimulating Factor (M-CSF), Granulocyte-Macrophage Colony-Stimulating Factor (GM-CSF), Interferon-γ (IFN-γ), Tris buffered saline, Glycine, Goat serum, Tween-20, Tartrazine. Enzyme-linked immunosorbent assay (ELISA) Duoset kits for human IL-4, TNF-α, IL-6, CCL-18, and CCL-22 were purchased from R&D systems. Human TGF-β1 Pre-Coated ELISA Kit (BGK01137) and Recombinant Human TGF-β1 (100-21C) were purchased from PeproTech™. The following materials were purchased from Thermo Fisher Scientific: Pierce™ Detergent compatible Bradford Assay, RPMI 1640 cell culture media, GlutaMAX™, Glucose solution, HEPES buffer (4-(2-hydroxyethyl)-1-piperazineethanesulfonic acid), Sodium pyruvate, Trypan blue, Antibiotic-Antimycotic, Calprotectin Mouse Anti-Human (27E10), Goat anti-Mouse IgG (H + L) Cross-Adsorbed Secondary Antibody, Rhodamine Red™-X (R-6393), Goat anti-Rabbit IgG (H + L) Cross-Adsorbed Secondary Antibody, Alexa Fluor™ 488 (A-11008), DAPI (4′,6-Diamidino-2-Phenylindole, Dihydrochloride), Thermo Scientific™ 1-Step Ultra TMB-ELISA Substrate Solution, Sulfuric acid, isopropanol. Anti-Mannose Receptor anti-human antibody (ab64693) was purchased from Abcam. ToxiLight™ Non-Destructive Cytotoxicity BioAssay Kit (LT17-217) and 100% lysis Control set (LT07-517) were purchased from Lonza™.

### Hydrogel UV casting

Hydrogel precursor solutions were prepared in PBS while protected from light. 0.5% w/v Irgacure-2959 was dissolved at 70 °C for 20 minutes. Acrylate monomers and proteins of interest were dissolved at room temperature (RT). Hydrogels were photocrosslinked by 10 minutes 365 nm UV light exposure under argon with oxygen concentration below 2000 ppm. 500 μL hydrogels were cast in 15 mm × 15 mm square poly(tetrafluoroethylene) (PTFE) moulds and 100 μL hydrogels were formed as droplets on a flat piece of PTFE.

### *In vitro* hydrogel swelling

Hydrogels were immersed in 6 mL PBS at 37 °C and weighed multiple times over a 5 day period. The swollen weight was expressed as a percentage increase from the initial hydrogel weight immediately after UV photocrosslinking.

### Hydrogel compressive characterisation

Compressive modulus was characterised by compressing swollen hydrogels using a texture analyser (Stable bio systems TA·HD plus) with a 5 kg load cell. Hydrogels were compressed at 0.1 mm s^−1^ until 50% strain. The compressive modulus was calculated as the gradient of the linear section from the stress–strain curve.

### Scanning electron microscopy

10% w/v PEGDA hydrogels containing 0%, 1%, and 5% w/v SPAK were frozen overnight at −80 °C and subsequently freeze-dried for 24 hours (ModulyoD). Each sample was mounted on an aluminium stub using conductive carbon adhesive tape and sputter-coated with gold under argon atmosphere for 40 seconds at a current of 30 mA. Samples were placed in a FEI XL30 SEM under high vacuum and the working distance was optimised for each sample. Hydrogels were imaged at 10 kV.

### *In vitro* protein release (protein loading pre photocrosslinking)

Protein loading into the hydrogel precursor solution before photocrosslinking was used for lysozyme, TGF-β1, and IL-4 release in Fig. 2 and 3. Stock solutions of TGF-β1 or IL-4 were diluted into the hydrogel precursor solution to reach the desired final concentration for each experimental condition. Lysozyme was dissolved into hydrogel precursor solutions at 10 mg mL^−1^ which contained 10% w/v 575 Da PEGDA and 0%, 5%, 10% or 15% w/v SPAK. Following UV photocrosslinking, hydrogels were individually immersed in 6 mL in PBS for *in vitro* protein release at 37 °C. At each time point, the entire release buffer was collected and replaced. Supernatant collected from each time point was stored at −20 °C until the appropriate time for analysis by Bradford assay or ELISA.

### Bradford assay

Bradford assay was carried out according to supplier's instructions for the Micro-Microplate protocol. Briefly, lysozyme standards with 1.25–25 μg mL^−1^ concentrations were prepared in PBS. 150 μL of each standard or sample was mixed with 150 μL of Bradford reagent in a 96-well assay plate. Plates were incubated at room temperature for 10 minutes. Absorbance was read at 595 nm using a plate reader (TECAN infinite 200 PRO). A standard curve was used to calculate the lysozyme concentration in controlled release samples (ESI Fig. S1†). Samples measuring above the assay range were diluted in PBS and assayed again until they were in the linear dynamic range of the standard curve.

### Lysozyme bioactivity assay

Micrococcus lysodeikticus was dissolved in PBS at a concentration of 0.15 mg mL^−1^. For each lysozyme controlled release time point to be tested, a control sample of fresh lysozyme was prepared in PBS with a matched concentration to the controlled release sample. Lysozyme bioactivity was determined using a UV-Vis spectrophotometer (Agilent Cary UV-Vis MulticellPeltier). 1.5 mL of lysozyme substrate was pipetted into a disposable cuvette followed by 100 μL of either controlled release supernatant or control solution. Cuvettes were mixed once by immersion and immediately placed into the spectrophotometer chamber. The absorbance was read at 450 nm and then read again every minute for 5 minutes. The average decrease in absorbance in 5 minutes for controlled release samples was expressed as a percentage of the decrease in its matched control.

### *In vitro* IL-4 sustained release (IL-4 loading post photocrosslinking)

Protein loading post photocrosslinking was used for sustained IL-4 release (Fig. 4). Hydrogel precursor solutions were prepared in PBS containing 10% w/v 4 kDa PEGDA and 0%, 1% and 5% w/v SPAK. Hydrogels were photocrosslinked using 10 minutes 365 nm UV light exposure under argon with O_2_ < 2000 ppm. Hydrogels were individually washed with 5 mL PBS at 37 °C for 5 days. Hydrogels were sterilised using 254 nm UV light irradiation for 20 minutes (Benchmark UV-Clave Ultraviolet Chamber) followed by a 30 minute wash in antibiotic antimitotic and three PBS washes. Each gel was soaked in 500 μL of a 2.2 μg mL^−1^ IL-4 solution at 37 °C for 24 hours.

Hydrogels were then individually immersed in 2 mL of THP-1 complete cell culture media for *in vitro* release which consisted of RPMI 1640 supplemented with 10% FBS, 1% sodium pyruvate, 1% GlutaMAX™, 1% HEPES, 1.25% glucose solution and 1% penicillin–streptomycin. Media was completely changed at 2 hours, 4 hours, and 6 hours followed by once per day until day 5. Time points were then staggered to every 2 days until day 9, every 3 days until day 33 and then every 5 days until day 73. For each time point 1 mL of the collected supernatant was stored at −20 °C for macrophage polarisation. The remaining 1 mL was assayed by ELISA to quantify the IL-4 released at each time point.

IL-4 loading was estimated as the difference in IL-4 dose before and after 24 hours incubation with each hydrogel. A control group was included containing 5% SPAK-10% PEGDA which was incubated for 24 hours in PBS containing 1 mg mL^−1^ BSA with no IL-4.

### Enzyme-linked immunosorbent assay (ELISA)

TGF-β1 ELISA was carried out according to supplier's instructions. For IL-4, TNF-α, IL-6, CCL-18, and CCL-22, ELISA plates were coated with capture antibody overnight on a rocker at RT. ELISA plates were washed with PBS containing 0.05% tween-20 and blocked with 1% w/v BSA for 1 hour at RT. Supernatant was thawed and brought to RT. Plates were washed and incubated with supernatant for 1 hour on a rocker at RT. Plates were washed and incubated with appropriately diluted detection antibody for 1 hour on a rocker at RT. Plates were washed and incubated with appropriately diluted streptavidin-HRP conjugate for 20 minutes while protected from light. Plates were washed and incubated with TMB substrate for 20 minutes while protected from light. Sulfuric acid was added as stop solution and absorbance was read at 450 nm with 570 nm reference wavelength using a platereader (Promega GloMax® explorer). The cytokine concentration in each condition was calculated using a 7 point standard curve.

### Macrophage polarisation

THP-1 cells were purchased from ATCC (ATCC TIB-202). THP-1 cells were differentiated into macrophages as previously described with some modifications.^32^ Briefly, cells were cultured in the concentration range of 200 000–1 000 000 cells per mL in THP-1 complete media consisting of RPMI-1640 supplemented with 10% FBS, 1% sodium pyruvate, 1% GlutaMAX™, 1% HEPES, 1.25% glucose solution and 1% penicillin–streptomycin. THP-1 cells were differentiated into macrophage-like cells using 50 ng mL^−1^ PMA for 6 hours at a cell density of 500 000 cells per mL. Cells were washed twice with PBS and were incubated in complete media for 24 hours at 37 °C. Supernatant from IL-4 controlled release time points was then used to promote M2-like polarisation. Control groups for M0 (50 ng mL^−1^ M-CSF) and M1 (20 ng mL^−1^ IFN-γ + 50 ng mL^−1^ GM-CSF) were included. An M2 control was also included with a matched concentration of fresh IL-4 to each controlled release time point to investigate IL-4 bioactivity after long-term release. Released IL-4 and the M2 controls were supplemented with 50 ng mL^−1^ M-CSF. After 6 days of culture in polarisation media, supernatant was collected for analysis.

### Toxilight™ bioassay

Macrophage viability was tested using the Toxilight™ bioassay at days 3 and 6 of culture. 50 μL of culture supernatant was collected at each time point. In a white assay plate, 10 μL of supernatant was mixed with 50 μL of adenylate kinase detection reagent. The plate was incubated at RT for 5 minutes and luminescence was read using a plate reader (Promega GloMax® explorer). A control group was included with M0 cells seeded at 500 000 cells per mL that were lysed after 6 days of culture using the Toxilight™ 100% lysis reagent. The luminescence of the lysed control was treated as the value for 100% dead cells. The luminescence in experimental groups was expressed as a percentage of the lysed control to calculate the percentage of dead cells in each experimental condition.

### Immunostaining

Cells were washed twice with PBS and then fixed with 4% paraformaldehyde for 15 minutes. Cells were washed 3 times with 0.2% Tween-20 in PBS and were blocked with 3% BSA and 1% glycine in PBS for 30 minutes. Cells were washed 3 times and then blocked with 5% goat serum in PBS for 30 minutes. Cells were incubated with appropriately diluted primary antibody overnight at 4 °C. Cells were washed 3 times and incubated with appropriately diluted secondary antibody for 1 hour at RT while protected from light. Cells were washed twice and then incubated with DAPI solution for 5 minutes at RT while protected from light. Cells were washed three times and viewed using a fluorescent cell imager (ZOE™, Bio-Rad).

### 3D printing

3D objects were designed on https://www.Tinkercad.com. A porous design was created measuring 11 mm × 11 mm × 0.6 mm with twenty five 1 mm × 1 mm pores arranged in a five-by-five grid, with each pore being separated by 1 mm from neighbouring pores and outer pores being 1 mm from the material periphery. A non-porous design was created as a cuboidal prism measuring 9.71 mm × 9.71 mm × 0.6 mm with an approximately equal volume to the porous design.

Slice parameters were set to 25 μm *z* thickness, 5 mm *Z* lift distance, 4 mm s^−1^*Z* lift speed and 6 mm s^−1^*Z* retract speed using Photon workshop software (Anycubic). Hydrogel precursor solutions were prepared in PBS containing 10 mg mL^−1^ lysozyme, 0.5% w/v LAP, 10% 575 Da PEGDA, 2% or 5% SPAK, and 0.0255% tartrazine while protected from light. Printing was carried out on a Photon Mono 4k printer (Anycubic) with 45 Watt power and a 30 second exposure time for the bottom two layers, followed by a 4 second exposure for the remaining 22 layers. Before and after each use the printer vat and platform were removed and cleaned with isopropanol. Porous hydrogels containing 2% SPAK and 5% SPAK were viewed by brightfield microscopy and images of 5 pores from 5 separate hydrogels were taken and measured using AMscope software.

### Statistical analysis

Hydrogel characterisation and controlled release data is presented as mean ± standard deviation (SD) with each experimental condition tested in triplicate. Cell culture data is presented as mean ± standard error of the mean (SEM) from two independent experiments with three experimental repeats per condition. Statistical analysis was carried out in GraphPad PRISM 9.0. One-way ANOVA was performed to determine significant differences between experimental conditions when three or more were present on a column graph (*α* = 0.05). Tukey's *post hoc* test was performed for pair-wise comparisons. Student's *t*-test was used to determine significant differences between the mean of two experimental groups. Pairs with significant differences were labelled as *P ≤ 0.05, **P < 0.01, ***P < 0.001 and ****P < 0.0001.

## Results

### Controlled lysozyme release from SPAK–PEGDA hydrogels

500 μL hydrogels were UV cast containing 10% w/v 575 Da PEGDA and 0%, 5%, 10%, and 15% w/v SPAK. SPAK was selected to electrostatically attract proteins with iso-electric points above 7.0 including IL-4 and TGF-β1.^33,34^

**Fig. 1:**
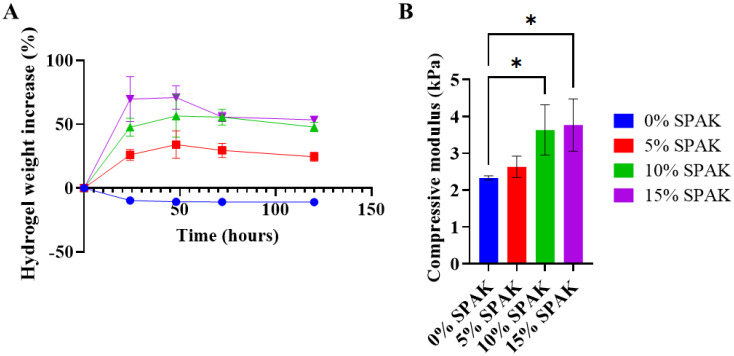
Characterisation of swelling and compressive modulus in UV cast SPAK–PEGDA hydrogels. (A) 5 Day *in vitro* swelling of 10% 575 Da PEGDA hydrogels containing 0%–15% SPAK. (B) Compressive modulus of swollen SPAK–PEGDA hydrogels. Values presented as mean ± standard deviation (*n* = 3). * denotes a statistical difference with *P* ≤ 0.05 determined by 1 way ANOVA with Tukey's *post hoc* multiple comparisons.

5-Day *in vitro* swelling showed SPAK to increase hydrogel swelling (Fig. 1A). Increased swelling is likely due to the anionic groups attracting water molecules. The decrease in weight exhibited by 0% SPAK-10% PEGDA is indicative of residual monomer diffusing out of the hydrogel (Fig. 1A). The average compressive modulus in all conditions were between 2–4 kPa which is comparable with previously reported values and indicates satisfactory crosslinking^35,36^ (Fig. 1B).

Initial protein release experiments used lysozyme as a low-cost model protein. Lysozyme was selected because its iso-electric point of 11.35 and molecular weight of 15 kDa are comparable to IL-4 and TGF-β1.^33,37–39^ A schematic of the protein release method is included (ESI Fig. S2†).

**Fig. 2:**
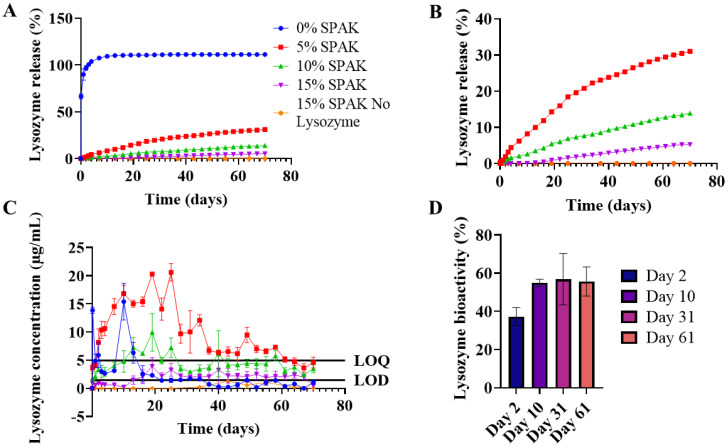
Controlled lysozyme release from SPAK–PEGDA hydrogels. 500 μL hydrogels were cast by UV photocrosslinking containing 10% w/v 575 Da PEGDA, 0%, 5%, 10%, or 15% SPAK, and 10 mg mL^−1^ lysozyme. Hydrogels were immersed in PBS for a 70 day controlled release study. (A) Effect of SPAK on lysozyme release. (B) Effect of SPAK on lysozyme release showing only 5–15% SPAK. (C) Concentration of lysozyme released at each time point, the limit of detection (LOD) and limit of quantification (LOQ) were 1.46 μg mL^−1^ and 4.94 μg mL^−1^. The first 7 time points of 0% SPAK–10% PEGDA were diluted to fit the assay range. (D) Bioactivity of lysozyme released from 5% SPAK–10% PEGDA at days 2, 10, 31, and 61 of *in vitro* release. Values presented as mean ± standard deviation, where error bars cannot be seen they are smaller than the marker at each plotted point. Bradford assay was performed in duplicate (*N* = 2, *n* = 3) and UV-vis spectrophotometry was performed in triplicate (*N* = 3, *n* = 3).

0% SPAK–10% PEGDA exhibited rapid burst release with a measured percentage release of 100% after 3 days (Fig. 2A). SPAK potently reduced burst release and had a dose dependent effect on controlling lysozyme release rate (Fig. 2A and B). 5% SPAK–10% PEGDA had zero order release for the first 31 days followed by a slight decrease in release rate until day 70, at which 31% of total lysozyme had been released (Fig. 2A–C). 10% SPAK–10% PEGDA had a linear release trend for 70 days with cumulative release of 13.9% (Fig. 2A–C). 15% SPAK–10% PEGDA exhibited delayed release and started to release detectable lysozyme after 13 days, and then had a linear release profile which reached 5.2% at day 70 (Fig. 2A–C).

A control group containing 15% SPAK–10% PEGDA with no lysozyme loading was included and had no response above the LOD at any time point which confirms there was no cross reactivity between the Bradford assay and residual monomer or hydrogel degradation products that may be in the release supernatant (Fig. 2A–C).

Lysozyme released from 5% SPAK–10% PEGDA had bioactivity of 37%, 55%, 57%, and 56% at days 2, 10, 31 and 61 of release (Fig. 2D). It is remarkable that lysozyme retained 56% bioactivity after 61 days *in vitro*. Bioactivity below 100% shows that the tertiary structure is damaged during the UV photocrosslinking reaction. When exposed to 10 minutes UV irradiation alone, lysozyme bioactivity did not decrease (ESI Fig. S3†).

This suggests free radicals from activated photoinitiator reduce bioactivity but that bioactivity is then maintained during long-term controlled release. Free radicals have previously been reported to damage proteins including lysozyme by inducing oxidative stress at the disulphide bridges responsible for protein folding. The addition of various anti-oxidant binding molecules has previously offered protection.^40^ As there is no deterioration in bioactivity on days 31 and 61, we believe lysozyme sequestration within SPAK–PEGDA retains bioactivity which is essential for future cytokine delivery applications (Fig. 2D).

### Scaling down of hydrogel volume and protein loading concentration

Hydrogel volume and loading concentration were scaled down with the aim to achieve controlled release of TGF-β1 and IL-4 in the ng mL^−1^ concentration range relevant for biological signalling.

**Fig. 3:**
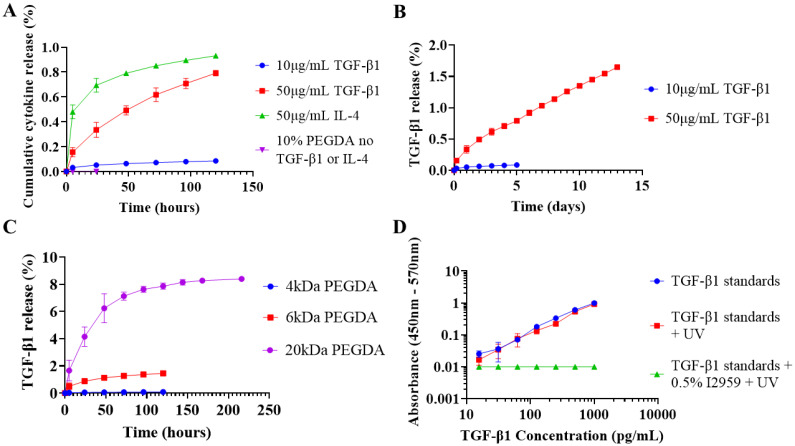
TGF-β1 and IL-4 release from PEGDA hydrogels. Following preliminary experimentation using lysozyme, hydrogel volume and loading concentration were decreased with the intention of releasing M2 promoting cytokines in the ng mL^−1^ concentration range. (A) TGF-β1 and IL-4 release from 100 μL 10% 4 kDa PEGDA. (B) 13 day TGF-β1 release from 10% 4 kDa PEGDA loaded with 50 μg mL^−1^ TGF-β1. (C) Effect of PEGDA molecular weight on 10 μg mL^−1^ TGF-β1 release. (D) Effect of UV photocrosslinking conditions on TGF-β1 quantification. Values presented as mean ± standard deviation, where error bars cannot be seen they are smaller than the marker at each plotted point. ELISA was performed in duplicate (*N* = 2, *n* = 3).

TGF-β1 loaded at 10 μg mL^−1^ in 100 μL 10% 4 kDa PEGDA exhibited exceptionally low cumulative release and was less than 0.1% after 5 days (Fig. 3A). 4 kDa PEGDA was selected because it showed improved total percentage release using 1 mg mL^−1^ lysozyme (ESI Fig. S4†). To increase release duration, TGF-β1 loading was increased to 50 μg mL^−1^ and the percentage release increased at every timepoint but was still below 2% after 13 days (Fig. 3B).

IL-4 was compared to TGF-β1 at 50 μg mL^−1^ and release was still less than 1% after 5 days which indicates the phenomenon was not cytokine specific (Fig. 3A). PEGDA molecular weight was increased to 6 kDa, and then to 20 kDa to increase mesh size which has previously facilitated protein diffusion.^41^ At 10 μg mL^−1^ loading, cumulative release increased to 1.5% in 6 kDa at day 5 and to 8.5% in 20 kDa at day 9 (Fig. 3C).

To investigate if crosslinking conditions were negatively affecting cytokine release, TGF-β1 standards were irradiated with 365 nm UV for 10 minutes both in the presence of Irgacure-2959 and without. No detectable response was seen in irradiated standards containing Irgacure-2959 and a colour change from colourless to white was observed, which could possibly be photocrosslinked protein aggregates (Fig. 3D and ESI Fig. S5†). The standard curve for irradiated standards not containing Irgacure-2959 was almost identical to the normal curve (Fig. 3D).

Photocrosslinking between proteins and expanding PEGDA chains would render a finite amount of protein permanently immobilised within the hydrogel and is a possible mechanism behind our low cumulative release. At low loading concentrations where the photoinitiator to protein ratio is high, the entrapped protein would represent the majority of the total and explain the low release we saw with 10 μg mL^−1^ TGF-β1 (Fig. 3A).

### Sustained IL-4 release from SPAK–PEGDA hydrogels

**Fig. 4:**
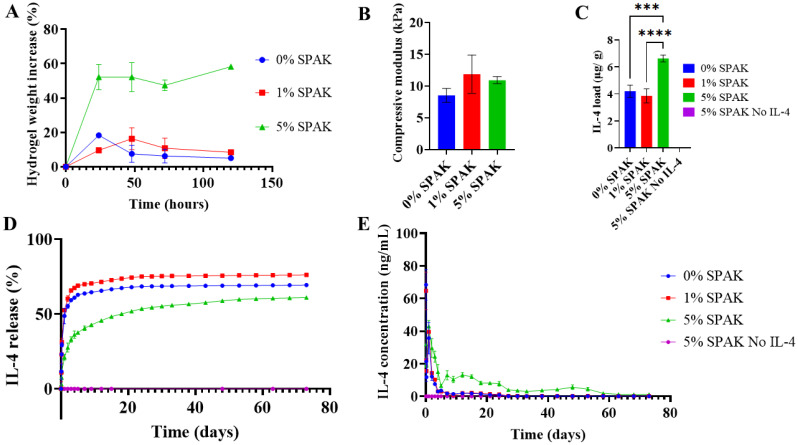
Sustained IL-4 release from SPAK–PEGDA hydrogels. (A) 5 day *in vitro* swelling of 10% w/v 4 kDa PEGDA hydrogels containing 0%, 1% and 5% SPAK. (B) Compressive modulus of swollen SPAK–PEGDA hydrogels. (C) Average IL-4 loading dosage per condition normalised to day 5 swollen hydrogel weight. (D) Cumulative IL-4 release from SPAK–PEGDA hydrogels. (E) IL-4 concentration released at reach time point. Values presented as mean ± standard deviation, where error bars cannot be seen they are smaller than the marker at each plotted point. ELISA was performed in duplicate (*N* = 2, *n* = 3). Average LOD and LOQ were determined as 31.1 pg mL^−1^ and 127.4 pg mL^−1^. Following one-way ANOVA and Tukey's *post hoc* multiple comparisons test, pairs with significant differences were labelled as *P ≤ 0.05, **P < 0.01, ***P < 0.001 and ****P < 0.0001.

Cytokine loading was modified to separate IL-4 from activated photoinitiator. A schematic of the modified method is included (ESI Fig. S6†). 100 μL hydrogels were UV cast with 10% 4 kDa PEGDA and 0%, 1%, and 5% SPAK. Hydrogel swelling was characterised in PBS for 5 days in which 0%, 1%, and 5% SPAK had 5.1 ± 4.1%, 8.5 ± 2.4% and 58.2 ± 1.5% respectively (Fig. 4A). The compressive modulus of swollen 0% SPAK, 1% SPAK and 5% SPAK hydrogels were 8.5 ± 1.1 kPa, 11.9 ± 3.0 kPa and 10.9 ± 0.6 kPa (Fig. 4B). Hydrogel pore size was increased in 5% SPAK–10% PEGDA which correlates with increased swelling (ESI Fig. S7†).

Hydrogels were immersed in a 2.2 μg mL^−1^ IL-4 solution for 24 hours. IL-4 loading dose was estimated as 4.20 ± 0.45 μg, 3.85 ± 0.52 μg, and 6.63 ± 0.26 μg for 0%, 1%, and 5% SPAK respectively. Increased IL-4 loading in 5% SPAK is likely due to greater electrostatic attraction between IL-4 and the polymer network. No increase in IL-4 loading was observed in 1% SPAK compared to 0% SPAK (Fig. 4B). It is possible in 1% SPAK that an insufficient amount of anionic groups are present at the hydrogel periphery to interact with IL-4 during the 24-hour loading period.

0% SPAK and 1% SPAK exhibited burst release, reaching 64% and 70% release after 7 days and both reaching a plateau after 24 days (Fig. 4D). We considered if the concentration of IL-4 released at each time point was similar to those commonly used for *in vitro* macrophage polarisation which are 10–20 ng mL^−1^.^42,43^ 0% SPAK–10% PEGDA released 68 ng mL^−1^ after 2 hours, 12 ng mL^−1^ at day 2, and then fell below 10 ng mL^−1^ at day 3 and was less than 1 ng mL^−1^ after 21 days for all remaining time points (Fig. 4E). 1% SPAK–10% PEGDA released 65 ng mL^−1^ after 2 hours, 14 ng mL^−1^ at day 2, fell below 10 ng mL^−1^ after 4 days, and was below 1 ng mL^−1^ from day 27 in all remaining time points (Fig. 4E).

Despite being loaded with significantly more IL-4, 5% SPAK–10% PEGDA released a lower concentration at the 2 hour time point with 32 ng mL^−1^, followed by sustained release in the ng mL^−1^ range. The concentration was 30 ng mL^−1^ at 2 days, at 18 days the concentration fell below 10 ng mL^−1^, and after day 53 the released concentration fell below 4 ng mL^−1^. Release remained above 1 ng mL^−1^ until day 73 with 61% total release (Fig. 4D and E).

### Promotion of M2-like macrophage polarisation with long-term IL-4 release

To determine the bioactivity of IL-4 released from SPAK–PEGDA, supernatant from *in vitro* release time points was used to promote M2-like polarisation of THP-1 macrophages. Samples from 5% SPAK–10% PEGDA were selected because it was the only condition which sustained release in the ng mL^−1^ range for longer than one month. THP-1 macrophages were polarised with IL-4 from days 48 and 53 of *in vitro* release in two independent experiments. The IL-4 concentration at both time points was 4 ng mL^−1^. 50 ng mL^−1^ M-CSF was included to support macrophage differentiation.

**Fig. 5:**
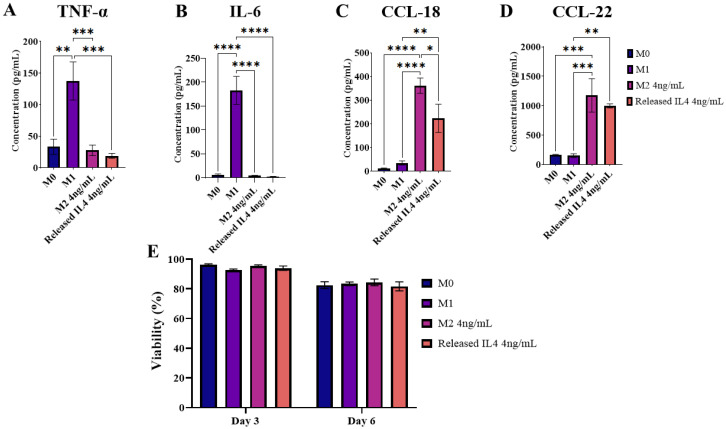
M2-like macrophage polarisation using IL-4 released from 5% SPAK–10% PEGDA. Four secreted polarisation markers were quantified by ELISA following six days of culture. TNF-α and IL-6 were selected as M1 markers (A and B). CCL-18 and CCL-22 were selected as M2a markers (C and D). Secreted markers were compared between Released IL-4 and an M2 control containing fresh IL-4 at an equal concentration of 4 ng mL^−1^. Control groups of M0 and M1 polarisation were included. (E) Macrophage viability at 3 and 6 days of culture. Data presented as mean ± standard error of the mean from two independent experiments which used IL-4 supernatant from days 48 and day 53 of *in vitro* release (*N* = 2, *n* = 3). Following one-way ANOVA and Tukey's *post hoc* multiple comparisons test, pairs with significant differences were labelled as *P ≤ 0.05, **P < 0.01, ***P < 0.001 and ****P < 0.0001.

Released IL-4 stimulated CCL-18 and CCL-22 secretion with comparable concentrations to the M2 control (Fig. 5C and D). Preliminary data showed extremely low IL-10 secretion from THP-1 macrophages in response to fresh IL-4 so it was deselected as a marker (ESI Fig. S8†). Released IL-4 did not stimulate TNF-α or IL-6 secretion (Fig. 5A and B) and macrophage viability was statistically the same between all conditions at days 3 and 6 of culture, with the general decrease at day 6 likely due to exhaustion of nutrients in the culture media (Fig. 5E). Taken together, the absence of M1 markers and the presence of M2 markers shows that media conditioned by IL-4 releasing 5% SPAK–10% PEGDA is not pro-inflammatory and that Released IL-4 had retained bioactivity after 53 days of *in vitro* release.

Bioactivity was also tested at 20 ng mL^−1^ with day 1 and 2 supernatant, and at 12 ng mL^−1^ with day 12 and 15 supernatant. Compared to M0 and M1 controls, all Released IL-4 conditions had low TNF-α and IL-6 secretion, and high CCL-18 and CCL-22 secretion which is consistent with day 48 and 53 data (ESI Fig. S9 and S10†).

**Fig. 6:**
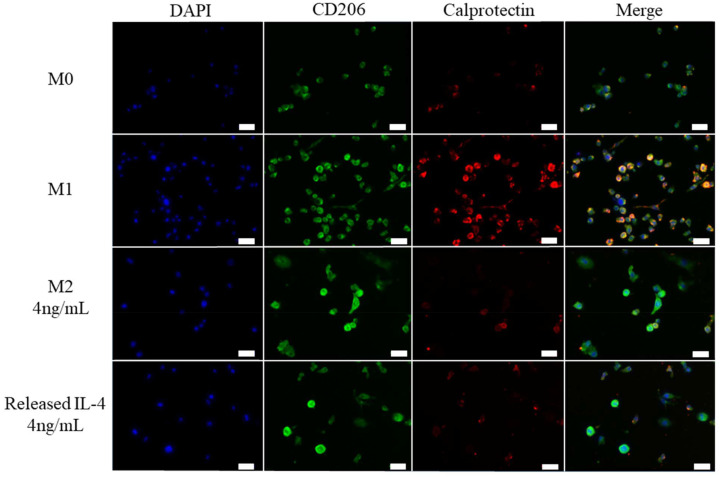
M1 and M2 surface markers in THP-1 macrophages. Day 53 IL-4 release supernatant from 5% SPAK–10% PEGDA was used to polarise THP-1 macrophages. Control groups for M0, M1 and M2 were included in which M2 had an equivalent dose of fresh IL-4 to the release supernatant. Calprotectin was chosen as an M1 surface marker, CD206 was chosen as an M2 surface marker. DAPI was used as a nuclear stain. Scale bar = 40 μm.

On day 6 macrophages were fixed and stained for M1 and M2 surface markers. Calprotectin was selected as an M1 marker and CD206 was selected as an M2 marker. M0 cells had low staining for CD206 and calprotectin (Fig. 6). M1 cells had high staining for calprotectin (Fig. 6). Both M2 4 ng mL^−1^ and Released IL-4 4 ng mL^−1^ had increased CD206 staining and low calprotectin compared to M0 and M1 controls which provides further evidence that IL-4 was bioactive at day 53 (Fig. 6).

### Lysozyme release from 3D printed SPAK–PEGDA hydrogels

The use of advanced manufacturing techniques to generate hydrogels with complex architectures has potential to encapsulate drugs in sophisticated structures for specialised biomedical applications.

To show proof-of-concept, we compared lysozyme release kinetics between hydrogels with two 3D designs that had differing surface area to volume ratios. SPAK–PEGDA hydrogels were 3D printed using digital light projection micro stereolithography containing 10 mg mL^−1^ lysozyme. Lysozyme was loaded pre-photocrosslinking because the release trend was previously more desirable (Fig. 2). *In vitro* release was compared between a non-porous cuboidal design and a design containing 25 pores with equal volume and 35% higher surface area to volume ratio.

**Fig. 7:**
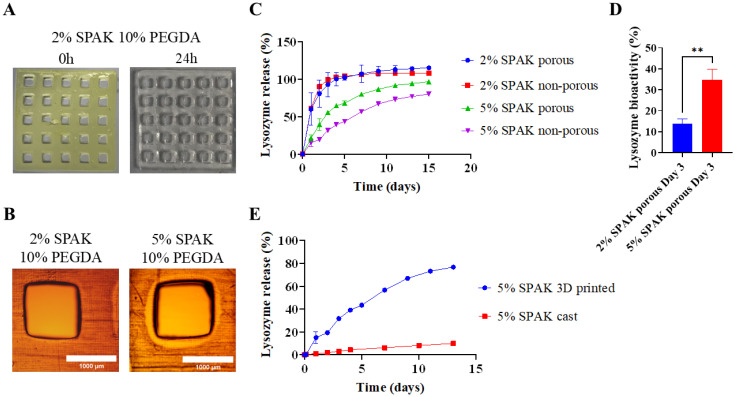
Lysozyme release from 3D printed SPAK–PEGDA hydrogels. (A) Macroscopic images of porous 3D printed 2% SPAK–10% PEGDA immediately after printing and after 24 hours. (B) Brightfield microscopy images of a representative macropore in 3D printed 2% SPAK–10% PEGDA and 5% SPAK–10% PEGDA (scale bar = 1000 μm). (C) 15 day lysozyme release from porous and non-porous SPAK–PEGDA. (D) Day 3 lysozyme bioactivity in porous 2% SPAK–10% PEGDA and 5% SPAK–10% PEGDA. (E) Lysozyme release from cast and 3D printed 5% SPAK–10% PEGDA. Values presented as mean ± standard deviation. Bradford assay was performed in duplicate (*N* = 2, *n* = 3). UV-vis spectrophotometry was performed in triplicate (*N* = 3, *n* = 3). Following unpaired Student's *t*-test ** denotes a statistical difference to *P* < 0.01.

Tartrazine used as photoabsorber visibly diffused out of the hydrogel after 24 hours (Fig. 7A). 2% SPAK–10% PEGDA had a measured pore diameter of 1110.7 ± 36.3 μm and 5% SPAK–10% PEGDA had a measured pore diameter of 1115.7 ± 35.4 μm (Fig. 7B and ESI Fig. S11†).

The release kinetics of lysozyme were compared over 15 days between porous and non-porous hydrogels containing 2% and 5% SPAK with 10% PEGDA. 5% SPAK–10% PEGDA had increased lysozyme release rate in the porous design compared to the non-porous, showing the increased surface area to enhance protein release (Fig. 7C). No noticeable difference in release rate was seen between porous and non-porous designs in 2% SPAK–10% PEGDA which suggests at low SPAK concentrations burst release occurs with similar kinetics regardless of 3D design (Fig. 7C).

Porous 2% SPAK–10% PEGDA and 5% SPAK–10% PEGDA had lysozyme bioactivity of 13.8 ± 2.3% and 34.8 ± 5.0% at day 3 (Fig. 7D). Increased electrostatic interaction between SPAK and lysozyme is likely to have preserved bioactivity in 5% SPAK–10% PEGDA.

Lysozyme release rate from 3D printed 5% SPAK–10% PEGDA hydrogels was increased compared to their cast counterparts (Fig. 7E). The differences in photoinitiator, UV exposure time, and that tartrazine was used in 3D printing mean that polymerisation efficiency is likely lower and less sulfate groups were present to control lysozyme release in 3D printed hydrogels.

## Discussion

This study sought to generate hydrogels exhibiting long-term controlled cytokine release that were synthetic, relatively low cost, and were able to be manufactured using advanced techniques. The material choice of SPAK–PEGDA was selected to form photocrosslinkable hydrogels containing negatively charged chemical groups. Photocrosslinked acrylate polymers also have a low biodegradation rate by hydrolysis which is essential for long-term controlled release applications.^44^

Our initial investigations used lysozyme as a cost effective model protein with similar molecular weight and iso-electric point to IL-4 and TGF-β1. At 10 mg mL^−1^ lysozyme loading, we have shown controlled protein release over a 70 day period with a dose dependent control over release rate (Fig. 2A–C). A controlled release period of 70 days is considerably longer than the 1–4 weeks seen in reports that have used electrostatic release methods with sulfated alginates and heparins.^16,19–21,45–47^ It is essential to achieve multiple months of controlled release so that medical outcomes in localised anti-inflammatory therapies and bone healing can be significantly improved.

Few protein release reports have shown a desirable release trend for multiple months *in vitro* and the longest reported protein release we are aware of showed 8 months delayed release of IgG.^13^ Such studies have utilised controlled material degradation and have often used bovine serum albumin which has no measurable bioactivity,^12,14,15^ or have not reported bioactivity data^13^ which severely limits their translatability into clinically relevant applications.

We have shown released lysozyme to have 56% bioactivity after 61 days which is remarkable after such a long period *in vitro*. We hypothesize that lysozyme bioactivity is reduced by photoinitiator in the photocrosslinking reaction and that the remaining bioactivity is maintained during sequestration within SPAK–PEGDA. Previous reports support our hypothesis, in which Irgacure-2959 was shown to reduce lysozyme bioactivity in similar conditions to our crosslinking reaction^48^ and when lysozyme bioactivity was maintained for 4 weeks in ionic liquids containing ethyl sulfate and PEG at temperatures of 25–45 °C.^49,50^ Given the chemical composition of our hydrogels, we expect that similar protective interactions occurred between lysozyme, sulfate groups on SPAK, and the PEG chains of PEGDA to maintain lysozyme bioactivity. We also observed higher bioactivity at days 10, 31, and 61 in comparison to day 2 which suggests lysozyme released at later time points had stronger electrostatic association with SPAK to maintain bioactivity, and that lysozyme released on day 2 had weaker association and lower bioactivity (Fig. 2D). Bioactivity may be further improved using antioxidant binding molecules as previously reported.^40^

The extrapolation of data collected in systems using high concentrations of model proteins into clinically relevant systems is challenging. In our work, we have shown that scaling down hydrogel volume from 500 μL to 100 μL, and reducing protein loading from the mg mL^−1^ to μg mL^−1^ range was met with profoundly low percentage release that was subsequently difficult to optimise (Fig. 3). Protein-photoinitiator interactions during photocrosslinking and subsequent protein-polymer interactions must be considered when attempting to control the release of immunomodulatory cytokines in biologically relevant doses. Systems must therefore be individually optimised for each material and cytokine combination and systems using high concentrations of model proteins will have limited relevance.

In our initial protein loading method, we saw evidence of photocrosslinking taking place in TGF-β1 standards in the presence of irgacure-2959 and UV irradiation when a colour change from colourless to white was seen and diminished ELISA detection was observed (Fig. 3D and ESI Fig. S5†). We believe these to be photocrosslinked aggregates of TGF-β1 and BSA. If so, these proteins would be expected to chemically conjugate to expanding PEGDA chains during the hydrogel photocrosslinking reaction. The entrapment of a finite amount of protein to the polymer network would explain why at the low protein loading concentrations of 10–50 μg mL^−1^ there is dramatically reduced percentage release (Fig. 3A–C) but that it is not observed at high concentrations like 10 mg mL^−1^ where the protein to photoinitiator ratio is higher (Fig. 2A).

Few publications have considered the interaction between proteins and photoinitiators during photocrosslinking. Lin *et al.* reported reduced lysozyme bioactivity and fractional release from PEGDA.^48^ They found using the more hydrophobic Irgacure-651 to increase lysozyme release and bioactivity compared to Irgacure-2959. They hypothesized that Irgacure-651 localises to the hydrophobic acrylate groups whereas Irgacure-2959 localises to the hydrophilic environment in close proximity to lysozyme and the PEG chains. The same research group also reported incomplete release of BSA from PEGDA and showed that the addition of a BSA binding ligand, iminodiacetic acid, improved cumulative release and that this effect was further enhanced in the presence of copper.^17^ Whether a similar binding ligand could have improved our TGF-β1 or IL-4 release remains a possibility.

We speculate that thiol–ene reaction is taking place between thiol residues present on cysteine amino acids and the expanding PEGDA chains. BSA used as a carrier in dilute TGF-β1 solutions is reported to have a free thiol group on its surface meaning it can take part in thiol–ene.^51,52^ BSA may then exert a sequestering effect on TGF-β1 to render it entrapped within the hydrogel. While TGF-β1 and lysozyme do not have surface cysteines containing thiol groups,^53–55^ altered protein folding may render internal thiols available for thiol–ene. The reduced bioactivity we observed in lysozyme (Fig. 2D) supports the claim of altered 3D structure and this effect may have been more pronounced at lower loading concentrations where the photoinitiator to protein ratio is greater.

Modification of the protein loading method was performed to separate photoinitiator and IL-4. When loading SPAK–PEGDA hydrogels with IL-4 by immersion, we observed sustained release in the ng mL^−1^ concentration range for 73 days from 5% SPAK–10% PEGDA (Fig. 4D and E). This period of quantifiable release is considerably longer than previously reported affinity based cytokine and growth factor release methods using heparins and sulfated alginates.^16,19–21,45–47^ Previous reports were likely limited by the short *in vitro* half-life of alginate based hydrogels which makes them inappropriate for long-term cytokine release applications.

The concentration of released cytokine at controlled release time points is a parameter that is often underreported and is typically overshadowed by reporting of the release trend. We have shown that 4 ng mL^−1^ IL-4 from days 48 and 53 of *in vitro* release was able to promote an M2-like macrophage polarisation (Fig. 5 and 6). To our knowledge, this is the longest period of time that bioactivity has been confirmed following long-term release in an *in vitro* model of immunomodulation.

The biodegradation of SPAK–PEGDA must be considered in future biomedical applications. We aimed to demonstrate controlled protein release using electrostatic affinity and did not want material degradation to significantly affect our experimental release kinetics which is why we did not include biodegradable crosslinkers. Incorporation of a small amount of biodegradable acrylates could trigger delayed biodegradation after the period of controlled release is complete. Delayed degradation has been observed in photocrosslinked hydrogels before in which multiple degradable linkers were compared to achieve the delayed release of IgG.^13^

Lastly, we 3D printed SPAK–PEGDA using micro stereolithography. We manufactured hydrogels with porous architecture organised in a five-by-five grid to demonstrate high print fidelity (Fig. 7A and B). We saw enhanced lysozyme release from porous 5% SPAK–10% PEGDA compared to non-porous hydrogels with lower surface area to volume ratio, showing proof-of-concept for control of protein release using 3D printed design (Fig. 7C).

There are exceptionally few reports showing protein release from 3D printed materials and they have shown limited release data for 10 days or less.^21,56^ We saw reduced release rate of lysozyme over a 15-day period in 3D printed 5% SPAK–10% PEGDA hydrogels compared to 2% SPAK–10% PEGDA (Fig. 7C). The release period of 15 days is comparable to many protein release reports but is sub-optimal when considering the time frame for regenerative medicine applications. The combining of advanced manufacturing techniques with biologics including cells and proteins remains challenging but is a cutting edge aspect of the regenerative medicine and biomaterials fields.

## Conclusion

SPAK–PEGDA hydrogels have been shown as promising materials for controlled cytokine release applications. In a model system, we controlled lysozyme release for a 70 days with tuneable control of release rate. We highlighted the difficulties encountered in scaling down protein loading concentration to release M2-associated cytokines TGF-β1 and IL-4, in which percentage release was significantly diminished.

By loading IL-4 post photocrosslinking, we showed sustained release in the therapeutic ng mL^−1^ range relevant for biological signalling. IL-4 release supernatant from as late as day 53 was able to promote M2-like macrophage polarisation *in vitro* as a model of long term immunomodulation using anti-inflammatory cytokine release.

We 3D printed SPAK–PEGDA by micro stereolithography with porous and non-porous geometries as a proof-of concept for controlling protein release using 3D printed design. Our data shows the potential of SPAK–PEGDA hydrogels as 3D printable, long-term cytokine release materials that may accelerate the essential clinical translation of cytokine release products for regenerative medicine and immunomodulatory applications.

## Author contributions

Conceptualisation: GAL, JY, AMG. Controlled release and cell culture data collection: GAL. Writing and reviewing of manuscript: GAL, JY, AMG. Scanning electron microscopy: CC. All authors approved the final version of the manuscript.

## Conflicts of interest

The authors declare no conflict of interest.

## Supplementary Material

BM-013-D4BM01623H-s001

## Data Availability

Data for this article, including controlled release data, are available at Mendeley Data at https://data.mendeley.com/datasets/tszcy9wczj/1.
