# Supplementary material for: Long-term interleukin-4 release from 3D printable affinity hydrogels promotes M2-like macrophage polarisation in vitro
Source: Biomater Sci. 2025 Mar 28;13(9):2489–502. doi: 10.1039/d4bm01623h (PMC11951857; doi:10.1039/d4bm01623h)
Supplement: BM-013-D4BM01623H-s001 [file BM-013-D4BM01623H-s001.pdf]

Supplementary Information (SI) for Biomaterials Science.
This journal is © The Royal Society of Chemistry 2025

Long-term Interleukin-4 Release from 3D Printable Affinity Hydrogels Promotes M2-like Macrophage Polarisation In-vitro

George A. Loxley[1,2,3,4], Consuelo Coser[1,2,3], Amir M. Ghaemmaghami[2*], Jing Yang[1,3*]

[1]Division of Regenerative Medicine and Cellular Therapies, Biodiscovery Institute, School of Pharmacy, University of Nottingham, Nottingham NG7 2RD, United Kingdom

[2]Immunology & Immuno-bioengineering Group, School of Life Sciences, Faculty of Medicine and Health Sciences, University of Nottingham, Nottingham NG7 2RD, United Kingdom

[3]Biodiscovery Institute, School of Pharmacy, University of Nottingham, Nottingham NG7 2RD, United Kingdom

[4]Section of Immunology, School of Biosciences, University of Surrey, Guildford, Surrey, GU2 7XH

Keywords: Controlled release, 3D printing, Immunomodulation, Affinity hydrogel, Cytokine release

*Corresponding authors:

jing.yang@nottingham.ac.uk

amir.ghaemmaghami@nottingham.ac.uk

Supplementary data

Bradford assay standard curve

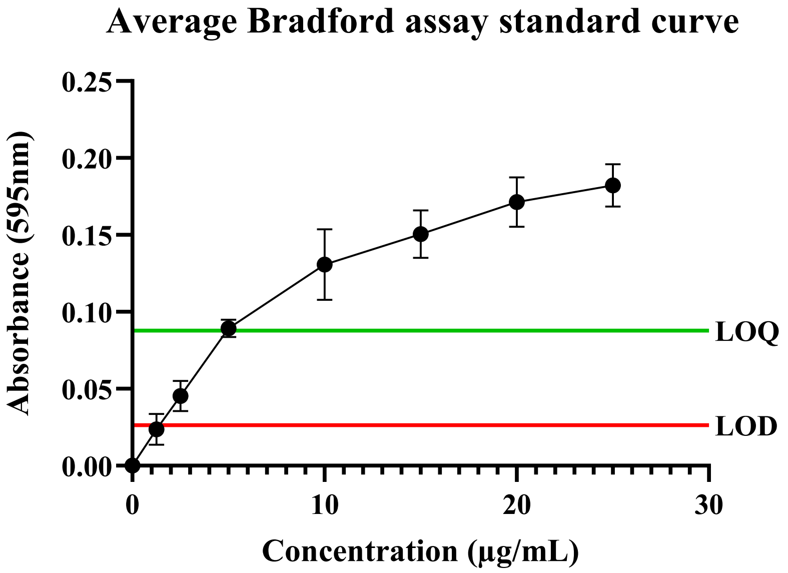

Figure S1: Average Bradford assay standard curve. Values presented as mean ± standard deviation (N=7, n=3).

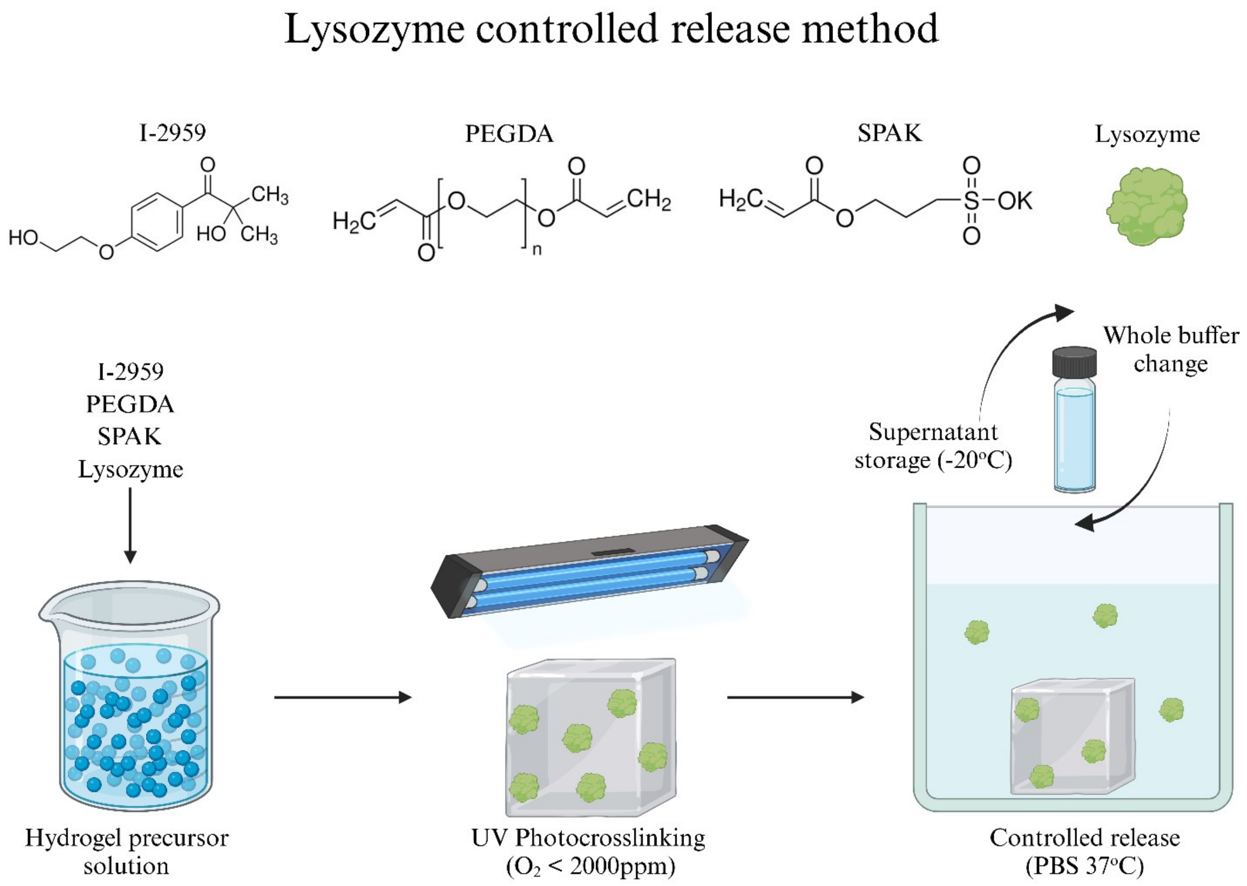

Figure S2: Schematic showing methodology for 70 day controlled release of lysozyme from SPAK-PEGDA

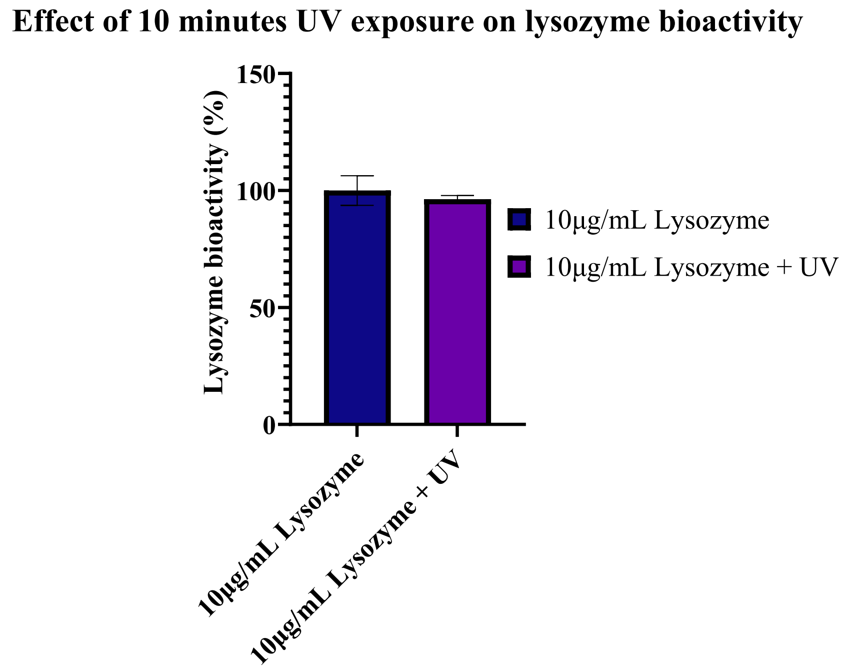

Figure S3: Effect of 10 minutes 365nm UV light irradiation on the bioactivity of 10µg/mL lysozyme. Values presented as mean ± standard deviation (N=3, n=3).

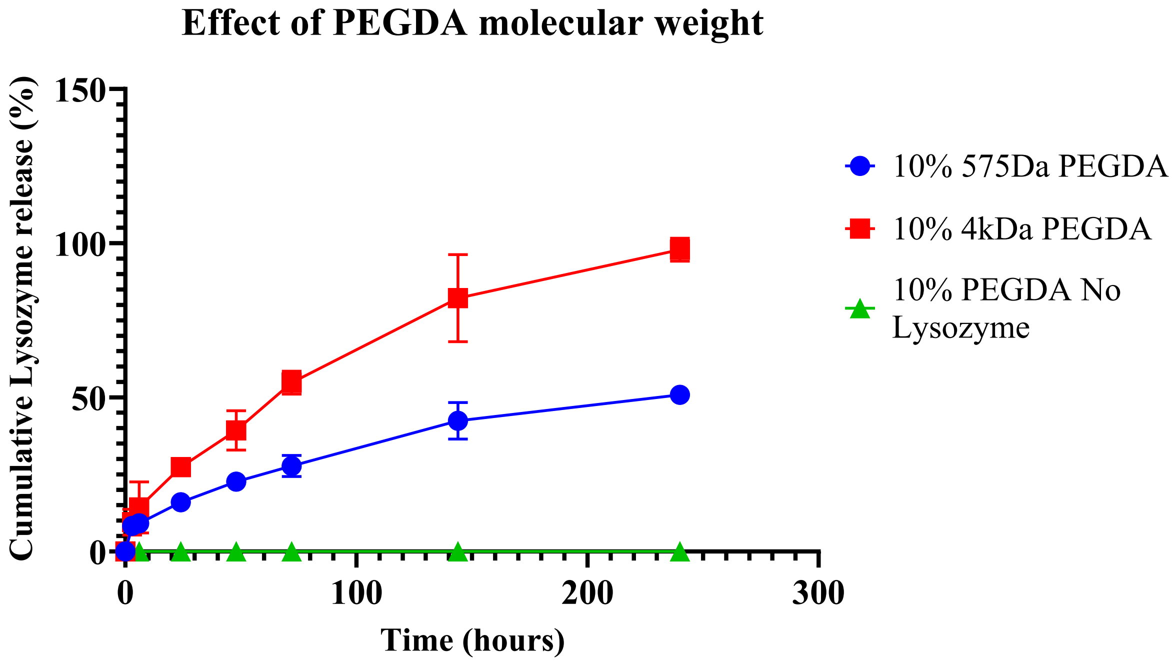

Figure S4 : Effect of PEGDA molecular weight on lysozyme release in 500µL 10% PEGDA hydrogels loaded with 1mg/mL lysozyme. Values presented as mean ± standard deviation (N=2, n=3).

<u>Effect of UV irradiation on TGF- β1 ELISA standards in the presence of 0.5% w/v irgacure-2959 photoinitiator</u>

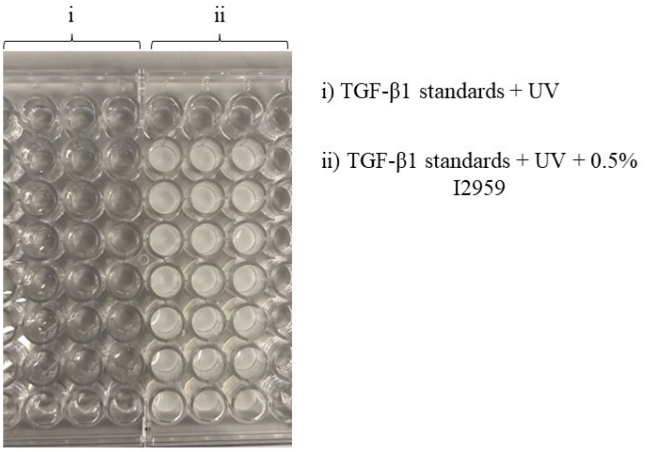

Figure S5 : Macroscopic image of TGF- β1 ELISA standards following 10 minutes 365nm UV light irradiation. i) TGF- β1 standards. ii) TGF- β1 standards containing 0.5% w/v Irgacure-2959 photoinitiator.

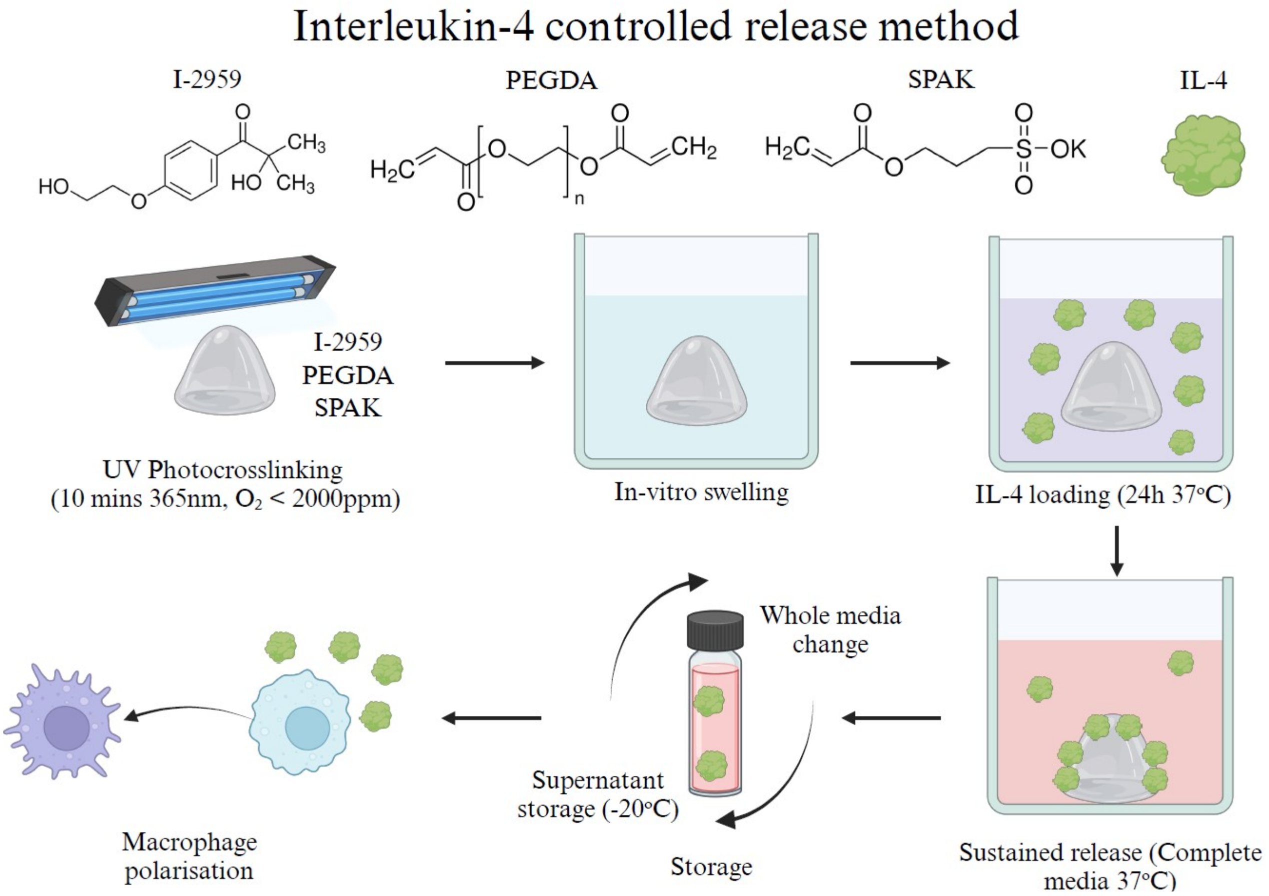

Figure S6: Schematic showing methodology for 73 day sustained release of Interleukin-4 from SPAK-PEGDA

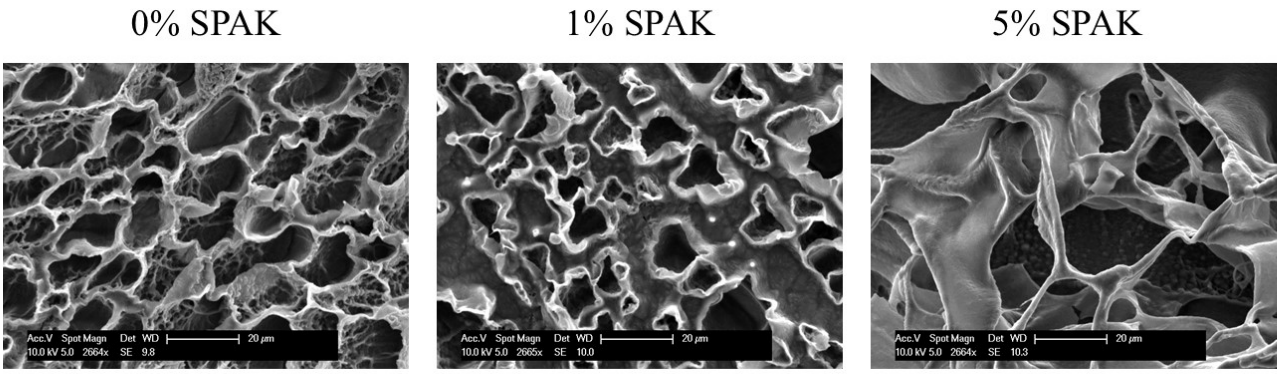

Figure S7: Scanning electron microscopy of 0%, 1%, and 5% SPAK-PEGDA hydrogels. Pore size was investigated in 10% 4kDa PEGDA hydrogels containing 0%, 1%, and 5% SPAK after 5 days of in-vitro swelling.

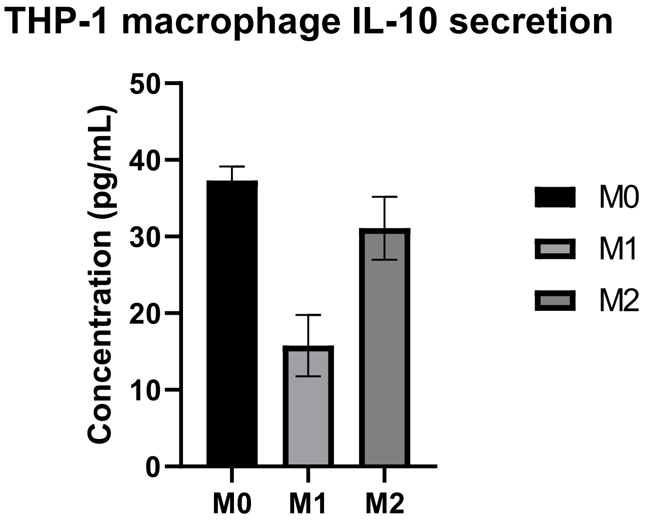

Figure S8: IL-10 secretion from THP-1 macrophages following 6 days of culture. M0 was polarised with 50ng/mL M-CSF, M1 was polarised with 50ng/mL GM-CSF + 20ng/mL IFN-γ, M2 was polarised with 50ng/mL M-CSF + 20ng/mL IL-4. Values presented as mean ± standard deviation (n=3).

<u>THP-1 macrophage polarisation using IL-4 controlled release supernatant from days 1 and 2 of in-vitro release from 5% SPAK 10% PEGDA</u>

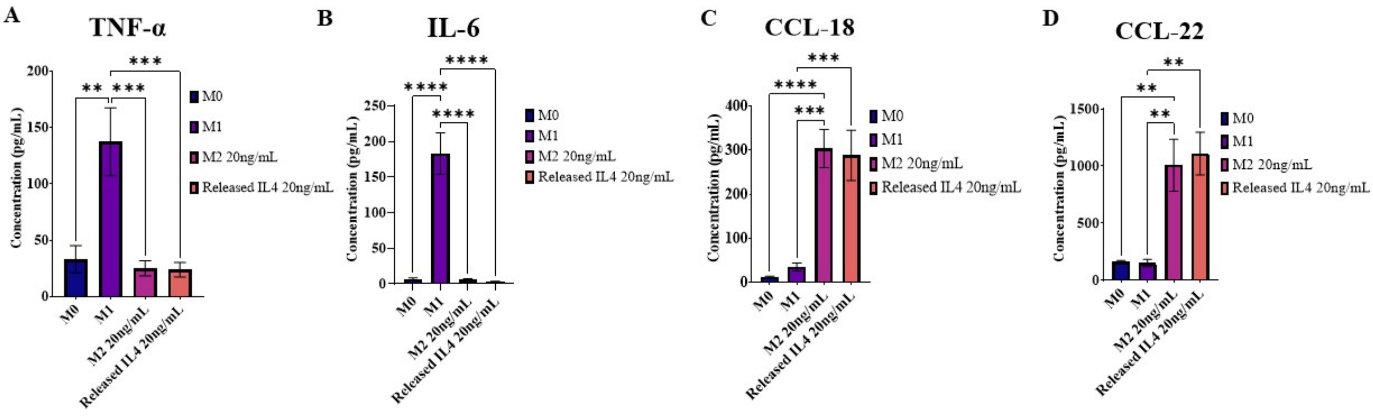

Figure S9: Secreted macrophage polarisation markers of THP-1 cells polarised with IL-4 released from 5% SPAK-10% PEGDA at days 1 and 2 of in-vitro release. TNF-α and IL-6 were selected as M1 markers. CCL-18 and CCL-22 were selected as M2 markers. Secreted markers were compared between released IL-4 and stock IL-4 at a concentration of 20ng/mL. Control groups of M0 and M1 polarisation were included. Data presented as mean ± standard error of the mean from two independent experiments which used media samples from day 1 and day 2 of controlled release respectively. (N=2, n=3). Following one-way ANOVA and Tukey's post hoc multiple comparisons test, pairs with significant differences were labelled as * P≤0.05, ** P<0.01, *** P<0.001 and **** P<0.0001.

THP-1 macrophage polarisation using IL-4 controlled release supernatant from days 12 and 15 of in-vitro release from 5% SPAK 10% PEGDA

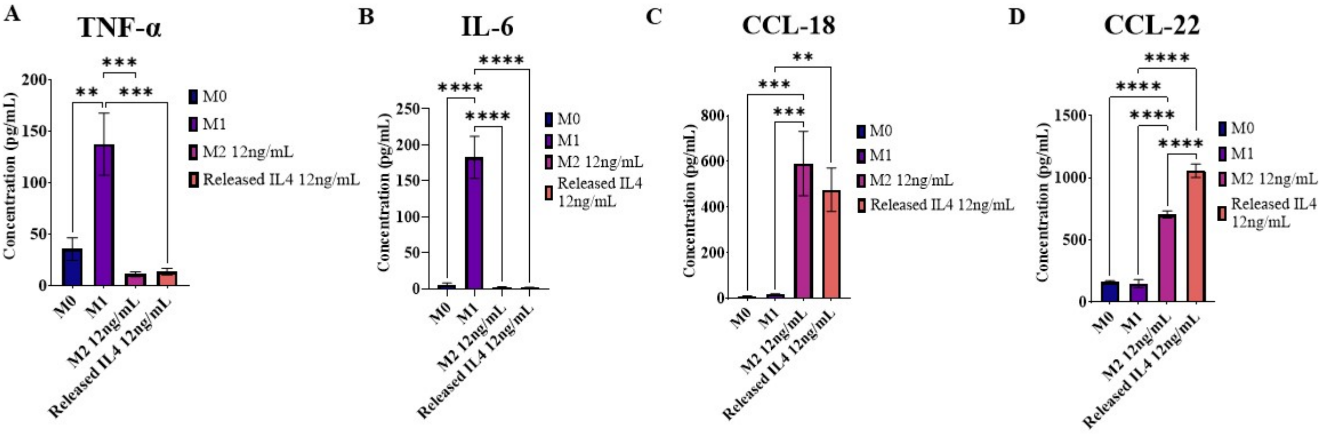

Figure S10: Secreted macrophage polarisation markers of THP-1 cells polarised with IL-4 released from 5% SPAK-10% PEGDA at days 12 and 15 of in-vitro release. TNF-α and IL-6 were selected as M1 markers. CCL-18 and CCL-22 were selected as M2 markers. Secreted markers were compared between released IL-4 and stock IL-4 at a concentration of 12ng/mL. Control groups of M0 and M1 polarisation were included. Data presented as mean ± standard error of the mean from two independent experiments which used media samples from day 1 and day 2 of controlled release respectively. (N=2, n=3). Following one-way ANOVA and Tukey's post hoc multiple comparisons test, pairs with significant differences were labelled as * P≤0.05, ** P<0.01, *** P<0.001 and **** P<0.0001.

Average diameter of macropores in 3D printed SPAK PEGDA hydrogels

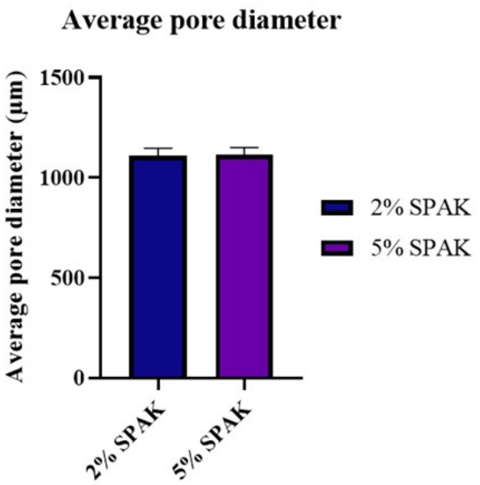

Figure S11: Average pore diameter from 3D printed SPAK PEGDA hydrogels. Mean calculated from measuring 5 pores from 5 separate hydrogels expressed as mean ± standard deviation (N=5 n=5).
